# Calculation of x-ray scattering patterns from nanocrystals at high x-ray intensity

**DOI:** 10.1063/1.4958887

**Published:** 2016-07-13

**Authors:** Malik Muhammad Abdullah, Zoltan Jurek, Sang-Kil Son, Robin Santra

**Affiliations:** 1 Center for Free-Electron Laser Science, DESY, Notkestrasse 85, 22607 Hamburg, Germany; 2 The Hamburg Centre for Ultrafast Imaging, Luruper Chaussee 149, 22761 Hamburg, Germany; 3 Department of Physics, University of Hamburg, Jungiusstrasse 9, 20355 Hamburg, Germany

## Abstract

We present a generalized method to describe the x-ray scattering intensity of the Bragg spots in a diffraction pattern from nanocrystals exposed to intense x-ray pulses. Our method involves the subdivision of a crystal into smaller units. In order to calculate the dynamics within every unit, we employ a Monte-Carlo-molecular dynamics-*ab-initio* hybrid framework using real space periodic boundary conditions. By combining all the units, we simulate the diffraction pattern of a crystal larger than the transverse x-ray beam profile, a situation commonly encountered in femtosecond nanocrystallography experiments with focused x-ray free-electron laser radiation. Radiation damage is not spatially uniform and depends on the fluence associated with each specific region inside the crystal. To investigate the effects of uniform and non-uniform fluence distribution, we have used two different spatial beam profiles, Gaussian and flattop.

## I. INTRODUCTION

With the advent of x-ray free electron laser (XFEL) sources,^1^ studies of structural determination of biomolecules^2–5^ have gained a new boost. XFELs provide intense radiation of a wavelength comparable to atomic scales. The characteristics of XFEL radiation and associated sample environments have triggered the development of new data collection methods such as serial femtosecond crystallography^6^ (SFX). The ultimate goal and dream is to perform atomic resolution single particle imaging.^7–11^ Sample damage by x-rays and low signal to noise ratio at high photon momentum transfer limit the resolution of structural studies on non-repetitive structures such as individual biomolecules or cells.^7,12^ Therefore, at high resolution, SFX is currently still a better option to use. XFELs deliver intense femtosecond pulses that promise to yield high-resolution diffraction data of nanocrystals (∼200 nm to 2 *μ*m in size) before the destruction of the sample by radiation damage.^13,14^ In SFX, a complete data-set can be obtained by exposing thousands of randomly oriented, individual crystals of proteins to the x-ray beam.

For imaging proteins and viruses at atomic resolution, one calls for high intensity and short x-ray pulses.^7,15–19^ The shortcoming of high intensities is the rapid ionization of the atoms on the few femtosecond timescale, which affects the structure of the system. This radiation induced damage changes the atomic form factors^20,21^ and may induce significant atomic displacement on longer times. Finally, radiation damage changes the scattering pattern. For a comprehensive theoretical study of signal formation in an SFX experiment, one needs to simulate (i) the radiation induced dynamics of the sample and (ii) pattern formation based on the dynamics. During the past decade, several models have been developed for studying the time evolution of small and large samples irradiated by XFEL pulses.^22–30^ We use XMDYN,^31,32^ a Monte-Carlo molecular-dynamics based code developed by the authors. In the theoretical study presented here, we consider a micron-size crystal in a 100 nm focus beam, a scenario where a nanocrystalline sample experiences fluences as high as to be used in single particle imaging experiments. As a consequence, the x-ray fluence is non-uniform throughout the sample. This may also have its imprint in the scattering pattern. The bottleneck one faces is that it is computationally not feasible to simulate a system with realistic size using tools which are capable to follow the dynamics of each atom, required for imaging studies. Therefore, we present an approach that involves the division of a crystal into smaller units (super-cells) and the calculation of their dynamics individually using periodic boundary conditions (PBC). In order to investigate the effect of inhomogeneous spatial fluence distribution, the super-cells are subjected to different fluences. Then we combine all the super-cells to form a nano-crystal and construct the scattering pattern under the influence of uniform (within the irradiated part of the sample) and non-uniform spatial beam profiles. We study and compare these two scenarios.

## II. METHODOLOGY

### A. Radiation damage simulation

XMDYN^31–33^ has been originally developed for modeling finite-size systems irradiated by an XFEL pulse. It unites a Monte-Carlo description of ionizations with a classical molecular-dynamical treatment of particle dynamics. XMDYN keeps track of the configuration of the bound electrons in neutral atoms and atomic ions. These configurations change dynamically because of different atomic processes like inner and outer-shell photoionization, Auger and fluorescence decay and collisional (secondary) ionization.

In order to treat x-ray-atom interactions, XMDYN uses the XATOM^21,34^ toolkit, which is an *ab-initio* framework based on non-relativistic quantum electrodynamics and perturbation theory. XATOM provides rates and cross-sections of x-ray-induced processes such as photoionization, Auger decay, and x-ray fluorescence. XMDYN employs XATOM data, keeps track of all the ionization events along with the electron configuration of each atom, calculates impact ionization and recombination, and follows the trajectories of all the ionized electrons and atoms solving the classical equations of motion numerically. The framework based on these microscopic processes can describe complex many-body phenomena in ionized systems such as nanoplasma formation, charge screening, thermalization of electrons through collisions and thermal emission.^32^ In the current study, chemical bonds between carbon atoms are not considered. This is a good approximation when the fluence is high enough to cause severe ionization in the system early in the pulse. The immediate ionization of the atoms leads to fast bond breaking that allows their exclusion in simulations.^31^

### B. Super-cell approach

The dimensions of the interaction volume are defined by the intersection of the x-ray beam and the crystal, therefore, its dimensions are determined by the focal area (∼100 × 100 nm^2^) and the thickness of the crystal along the beam propagation direction (*μ*m). The number of atoms within this volume is of the order of 10^9^. This number is formidably large: it is not feasible to simulate the whole system by a single XMDYN run. In order to overcome this barrier, we propose the procedure of dividing the whole crystal into smaller units. These super-cells may contain several crystallographic unit cells. We follow the dynamics within each super-cell driven by the local fluence (assumed to be uniform throughout the super-cell) individually. For this purpose, we have developed an extension to XMDYN that applies PBC^35,36^ to a super-cell, accounting also for the effect of the environment surrounding it.

Within the concept of PBC, a hypothetic crystal is constructed as a periodic extension of a selected super-cell. The total Coulomb interaction energy for a super-cell includes all the interactions within the given cell as well as pair interactions when one particle is in the cell while the other is in a periodic image within the super-cell based hypothetic lattice (PBC-crystal). Formally,

$$E=\frac{1}{4\pi \varepsilon_{0}}\frac{1}{2}\sum_{n}\sum_{i=1}^{N}{\sum_{j=1}^{N}}^{\prime }\frac{q_{i}q_{j}}{\left|r_{ij}+nL\right|},$$ (1)

where *N* represents the total number of particles in the super-cell, *q_i_* is the charge of the *i*th particle, *ε*_0_ is the dielectric constant, *L* represents the dimension of the cell (here assumed to be a cube), $nL=n_{1}c_{1}+n_{2}c_{2}+n_{3}c_{3}$, where $c_{1},c_{2},c_{3}$ represent basis vectors of the PBC-crystal, and *n*_1_, *n*_2_, *n*_3_ are integers indexing the periodic images. Hence, $\left|r_{ij}+nL\right|$ is the distance between the *i*th particle in the central super-cell (**n** = **0**) and *j*th particle in the super-cell indexed by **n**. The symbol ′ represents the exclusion of the term *j* = *i* if and only if **n** = **0**. The summation in Eq. (1) is not only computationally very expensive because of the formally infinite sum but is also conditionally convergent which states that the result depends upon the order of summation. To overcome this problem, we follow a route used often in the literature for spatially periodic systems, the method of minimum image convention.^37^ According to the convention: (i) when evaluating Eq. (1), we do not use the same super-cell division of the PBC crystal for all particles, but we always shift the boundaries so that the selected particle appears in the center; (ii) we consider only **n** = **0** terms. The former choice ensures that no jump happens in the potential energy when a particle crosses a super-cell boundary and therefore “jumps” in the evaluation from one border of the cell to the opposite. The latter is a minimum choice considering interactions between a selected particle with the closest copy of the others only. Finally, one can assemble the entire real crystal from the individually simulated super-cells to model the whole dynamics. While in this way modeling becomes feasible even without the need of super-computers, we should also note a shortcoming of the approach: we do not allow particle transport, in particular, electron transport between the super-cells. For biologically relevant light elements, Auger and secondary electrons have energies *E_kin_* ≲ 300 eV, which yields a short mean free path in a dense environment. Therefore, such electrons may travel only to neighboring super-cells experiencing similar fluences during the irradiation, so that the effect of net transport may be negligible. On the other hand, photoelectrons have an energy almost as high as the photon energy. Hence, they are fast and have a long mean free path: they can leave super-cells located at high fluences regions and can affect super-cells at larger distances experiencing lower fluences. We will overcome this shortcoming of the model in the future.

### C. Scattering intensity

Although during a single shot experiment the sample may undergo significant changes, the scattering patterns are static: they accumulate diffracted signal over the whole pulse. Further, the signal may contain an imprint of a spatially non-uniform intensity profile. Formally, the scattering intensity at a specific reflection described by reciprocal vector **Q**, including the integration over time and the subdivision of the crystal volume into super-cells according to the approach introduced in Section II B, reads

$$\begin{array}{c} \frac{dI(Q,F,\omega )}{d\Omega }=C(\Omega )\int_{-\infty }^{\infty }dt\;g(t)\sum_{I,r}P_{I,r}(F,\omega ,t)\\ {\left|\sum_{\mu }\sqrt{F_{\mu }}\;e^{iQ\cdot R_{\mu }}\;\sum_{X}\sum_{j=1}^{N_{X}}\;\;f_{X,I_{X,j}^{\mu }}(Q,\omega )\;e^{iQ\cdot r_{X,j}^{\mu }}\right|}^{2}. \end{array}$$ (2)

In this equation, **Q** is the momentum transfer, $F=\{F_{\mu }\}$ is the x-ray fluence distribution throughout the crystal, the index μ runs over all super-cells, and *ω* is the photon energy. *C*(Ω) is a factor depending the polarization of the x-ray pulse, and *g*(*t*) represents the normalized temporal envelope. $f_{X,I_{X,j}^{\mu }}$ is the atomic form factor of the *j*th atom of species *X* in the μth super-cell, $I_{X,j}^{\mu }$ is the associated electronic configuration, $I=\{I_{X,j}^{\mu }\}$ denotes a global electronic configuration, $r_{X,j}^{\mu }$ represents the position vector of the *j*th atom of species *X* in the *μ*th super-cell, and $r=\{r_{X,j}^{\mu }\}$ indicates the set of all atomic positions. *N_X_* represents the total number of atoms for species *X* within a super-cell. *P_I_*_,__*r*_ represents the probability distribution of electronic configuration *I* and atomic positions *r*, and **R**_*μ*_ represents the position of the μth super-cell. The atomic form factor

$$f_{X,I_{j}^{X}}(Q,\omega )=f_{X,I_{X,j}^{\mu }}^{\;0}(Q)+f_{X,I_{X,j}^{\mu }}^{\;\prime }(\omega )+i\;f_{X,I_{X,j}^{\mu }}^{\;\prime \prime }(\omega )$$ (3)

includes the dispersion corrections $f_{X,I_{X,j}^{\mu }}^{\;\prime }(\omega )$ and $i\;f_{X,I_{X,j}^{\mu }}^{\;\prime \prime }(\omega )$. This dispersion correction can be neglected when the applied photon energy is high above the ionization edges, which is fulfilled in our study. Note that the summation over $\sqrt{F_{\mu }}$ appears inside the modulus square in Eq. (2). The scattering amplitude from the μth super-cell is proportional to the x-ray field amplitude ($\propto \sqrt{F_{\mu }}$) in that super-cell. A key assumption when performing the coherent sum in Eq. (2) is that the entire crystal is illuminated coherently, a condition that is fulfilled considering realistic XFEL beam parameters and crystal sizes.

### D. XSINC: Scattering pattern simulation

In order to construct the scattering pattern, Eq. (2) cannot be used directly as the *P_I_* and *r* configuration space is too large. However, by calculating realizations of super-cell dynamics with XMDYN, a Monte-Carlo sampling of the distribution $P_{I,r}(F,\omega ,t)$ represented in Eq. (2) becomes feasible. To construct the time evolution of the crystal through global configurations and to calculate patterns, we used the following strategy, implemented in the code XSINC (x-ray scattering in nano-crystals).

**FIG. 1.**
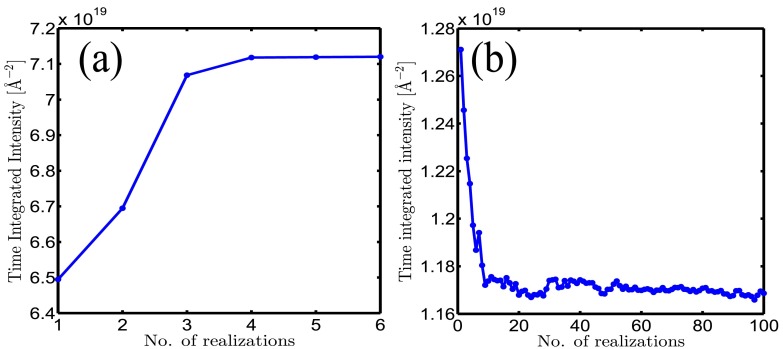
Convergence of time integrated peak intensity for the reflection (1 1 1) as a function of the number of realizations per fluence point: (a) for the Gaussian case and (b) the flattop case. For the Gaussian case, 350 different fluences points are used to calculate the time integrated intensity.

We discretize the fluence space and calculate many super-cell trajectories for each fluence value with XMDYN. XSINC selects randomly a trajectory for each super-cell within the crystal (a local realization), so that the corresponding fluence values are matching the best. These trajectories describe the local time evolution of the super-cells and together they form a global realization of the crystal. Then, taking into account the spatial and temporal pulse profiles, XSINC calculates the scattering amplitudes and intensities for the global configuration at different times based on the corresponding snapshots. Finally, the incoherent sum of these patterns corresponds to a time integrated pattern measured at in a single-shot experiment. In our calculation, we perform a dense sampling of the fluence space. As a consequence, two neighboring super-cells experience very similar fluence. Therefore, it is a good approximation to take into account the direct effect of the neighboring cells by applying periodic boundary conditions and this construction leads to a realistic global trajectory. In the scheme above, several parameters are convergence parameters of the method (Table I). Results are considered converged when characteristic properties of the Bragg peaks, such as the width and height of the intensity distribution in reciprocal space, converge during monotonic increase (or decrease) of the parameter. As an example, Figures 1(a) and 1(b) illustrate the convergence of the time integrated peak intensity as a function of the number of local (super-cell) realizations per fluence point for the reflection (1 1 1) for the Gaussian and flattop spatial profile cases. We note that convergence implicitly depends on the total number of different realizations used to build a global realization. Therefore, in the Gaussian case, where 350 different fluence points are used, convergence starts at a much smaller value.

**TABLE I.** Convergence parameters for calculating scattering intensity with XSINC and their values in the current study.

Convergence parameters	Gaussian case	Flattop case
Number of crystallographic unit cells in a super-cell	5 × 5 × 5	5 × 5 × 5
Number of fluence points	350	1
Number of local realizations (XMDYN trajectories) per fluence point	5	150
Number of assembled global realizations	10	10
Depth of the crystal in beam propagation direction	1 × Thickness of the super-cell lattice constant	1 × Thickness of the super-cell lattice constant
Number of snapshots	28	28

## III. RESULTS AND DISCUSSION

### A. Simulation setup

**FIG. 2.**
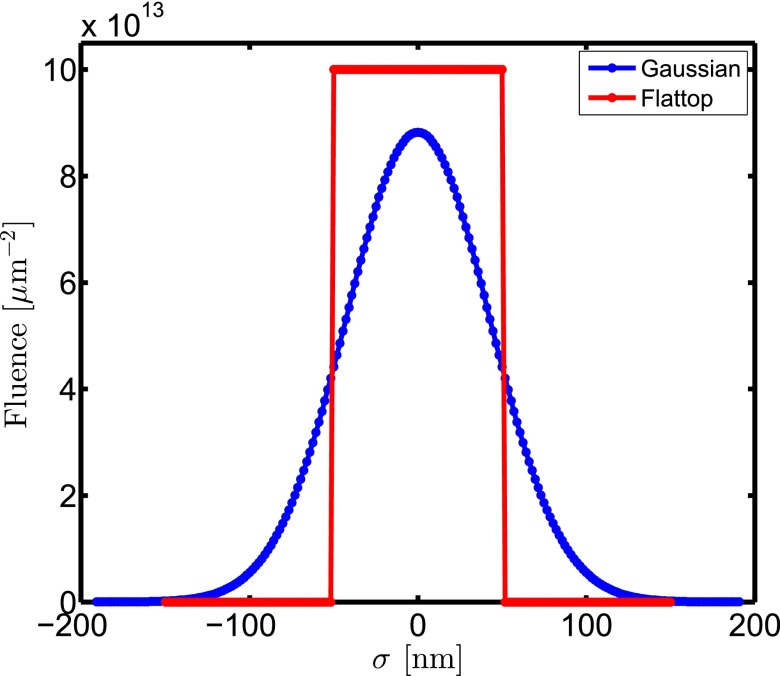
Radial fluence distributions in the current study: Gaussian profile (spatially non-uniform case) and flattop profile (uniform within the irradiated part of the crystal). The focal size is 100 nm in both cases, and the pulse energy is also considered to be same.

In our investigations, we consider a diamond cube of a size of 1 *μ*m. We investigate the cases of flattop and Gaussian beam profiles (Fig. 2). Other parameters of the pulses are the same in both cases: photon energy is 10 keV, total number of photons per x-ray pulse is 1 × 10^12^, the temporal pulse envelope is Gaussian with a duration of 10 fs FWHM, and focus size is 100 × 100 nm^2^ FWHM. The size of the diamond unit cell is *a* = *b* = *c* = 3.57 Å containing 8 carbon atoms. The parameter choices listed in Table I yield converged results.

### B. Radiation damage

**FIG. 3.**
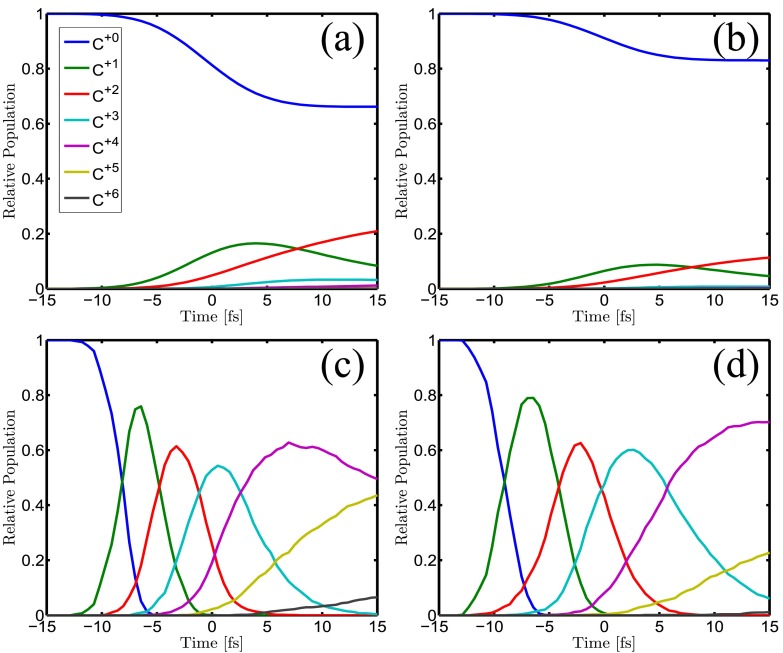
Ionization dynamics of carbon atoms at different fluences: time dependent charge state populations of isolated carbon atoms calculated with XATOM for (a) $F_{\text{high}}=1\times 10^{14}\;\mu m^{-2}$ and (b) $F_{\text{mid}}=4.5\times 10^{13}\;\mu m^{-2}$. Similarly, time dependent charge state populations of carbon atoms in diamond calculated with XMDYN for (c) $F_{\text{high}}$ and (d) $F_{\text{mid}}$. Secondary ionization events enhance the overall ionization in a dense environment. The x-ray pulse with 10 fs FWHM temporal profile is centered at *t* = 0 fs.

The coherent scattering patterns depend on the presence of the atomic bound electrons as well as on the atomic positions. The XMDYN and XATOM simulations allow to analyze their change due to radiation damage for both diamond and for the isolated carbon atom cases. Radiation damage is initiated by atomic photoionization events. In case of isolated carbon atoms, Auger decays contribute approximately to the same extent to the overall ionization. At the maximum fluence in our study, ∼35% of the atoms are photoionized (Fig. 3(a)). Although the absorbed energy is 10 keV per photon, almost all of this energy is taken away from the atom by the high energy photoelectron. The picture is different when the atom is embedded in a crystal environment (Fig. 3(c)). The high energy photoelectrons stay within the medium and distribute their energy by causing further ionization via secondary ionization events. As a consequence, neutral atoms disappear early in the pulse and by the end even fully stripped carbon ions (C^6+^) appear. Many electrons are promoted to (quasi-)free states within the sample. This also illustrates the importance of secondary ionizations in the progress of radiation damage in a dense environment.^38–40^ In the center of focus, the sample absorbs 3.5 keV energy per atom that heats up the plasma electrons beside the ionizations. Despite the high charge states, recombination remains negligible during the pulse (number of events less than 1% per atom in the simulation) due to the extreme conditions.

**FIG. 4.**
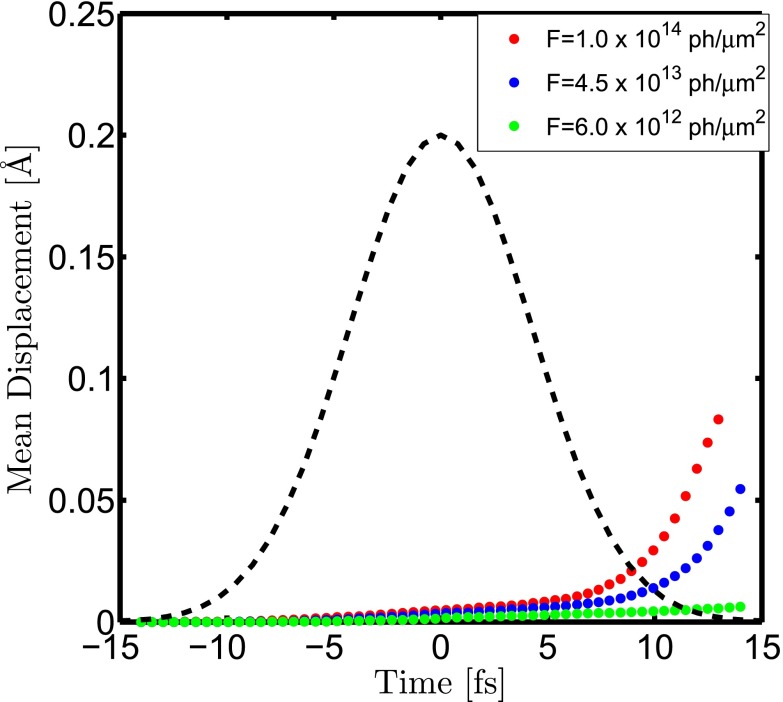
Mean displacement of the atoms for fluences $F_{\text{high}}=1\times 10^{14}\;\mu m^{-2}$ (red dots), $F_{\text{mid}}=4.5\times 10^{13}\;\mu m^{-2}$ (blue dots), and $F_{\text{low}}=6.0\times 10^{12}\;\mu m^{-2}$ (green dots). The Gaussian temporal pulse envelope is also depicted with the dashed black line. $F_{\text{high}}$ is the fluence for the flattop profile, which is also the maximum fluence in the present study. $F_{\text{mid}}$ and $F_{\text{low}}$ are two values representing intermediate and low fluences taken from the Gaussian profile case. The mean atomic displacement remains below the achievable resolution (∼1.2 Å) at 10 keV for all the cases.

Figure 4 represents the mean displacement of the carbon atoms during the pulse. The average atomic displacement is much below the maximum achievable resolution, ∼1.2 Å at 10 keV, even at the highest fluence. This suggests that the patterns are affected predominantly due to the bound-electron loss through the modification of atomic scattering form factors. Despite the heavy ionization, atomic displacements remain negligible during the ultrashort pulse duration due to the highly symmetrical sample environment. We note here again that in our calculations we neglected the chemical bonds. In low fluence regions bonds may survive and stabilize the structure against the emerging Coulomb forces. As the observed displacements are far below the resolution even without any stabilization due to bonds, bondless modeling of the current scenario is applicable.

*Effect of the PBC approach on the dynamics.* While ionic motion is negligible during the pulse, fast photoelectrons can travel long distances. However, PBC confines all plasma electrons artificially within the supercell they have been created in. Neglecting particle transport may lead to error in (*i*) local plasma electron density and (*ii*) local energy density. Whenever a photoelectron is ejected it leaves behind a positive charge located on an ion. If we consider Coulomb interaction only, a positive space charge would build up in a central cylinder because of photoelectron escape. Photoelectron trapping within the interaction volume would start early in the pulse, at an average ion charge as low as +0.005. An analogous phenomenon was discussed for finite samples in the literature.^29^ However, photoelectrons cause secondary ionization as well, so an atomic bound electron is promoted to a low energy continuum state. If this slow electron was created in an outer region, it can efficiently contribute to the screening of the space charge the photoelectron left behind. Based on these arguments we can conclude that (i) considering the interaction region to be neutral is a good approximation and (ii) in all regions we overestimate the energy density by confining fast photoelectrons within a supercell. Similarly, as the Coulomb forces are the driving forces of the ionic motions, we may also overestimate the atomic/ionic displacements. In our study eventually the effect on the scattering signal is relevant, as will be discussed in Sec. III C.

### C. Scattering with damage

**FIG. 5.**
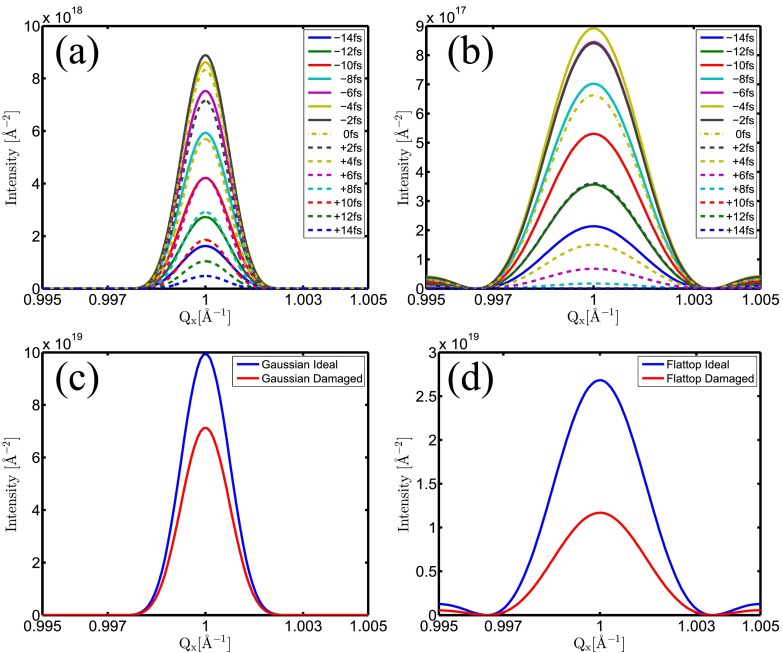
Snapshots of the scattering intensity for reflection (1 1 1) along the $Q_{y}=Q_{z}=1\;{Å}^{-1}$ line in reciprocal space: (a) Gaussian spatial beam profile, (b) flattop spatial beam profile. Solid and dashed lines with the same color correspond to the same instantaneous irradiating x-ray intensities. Note that the negative and the corresponding positive times are of equal intensity during the rise and fall of the pulse envelope. (c) and (d) Total time integrated scattering signal for Gaussian and flattop spatial beam profiles, respectively. Note the different vertical axis scales.

In this section, we analyze the changes of the Bragg peak intensity profiles in reciprocal space due to the severe radiation damage. In Figs. 5(a) and 5(b), snapshots of the 1D Bragg peak profiles in reciprocal space are depicted for the reflection *Q* = (1 1 1) for Gaussian and flattop spatial beam profiles, respectively. Two apparent features can be seen, valid for other reflections as well.

(i) The width of the Bragg peak does not change during the pulse. This is consistent with the expectation based on the negligible ion displacements: no visible Debye-Waller-like broadening occurs. However, the widths are different for the Gaussian and flattop cases. The reason is the difference between the size of the illuminated parts of the crystal. In the flattop profile case, the focus size defines strictly the region exposed. On the other hand, a Gaussian profile has no sharp edge and therefore illuminate a larger region, yielding a narrower Bragg peak and a larger effective crystal size.
(ii) Snapshots of the Bragg peak intensities behave differently for flattop and Gaussian beams. The snapshots of the Bragg intensities depend not only on the scattering power of the sample but also on the instantaneous x-ray intensity. However, as the instantaneous x-ray intensities are equal at the same time before and after the maximum of the pulse, a direct comparison of the corresponding snapshots of the Bragg profiles reflects exclusively the effect of different damage extents. In the Gaussian profile case, these corresponding curves show small difference only, indicating that a significant contribution is coming from regions in the crystal suffering little damage (Fig. 5(a)). In contrast, applying a flattop pulse profile, the scattering pattern is formed only from extensively ionized parts of the crystal. A consequence of the loss of atomic bound-electrons is the decrease of the atomic form factors yielding significant signal drop for longer times (Fig. 5(b)).

**FIG. 6.**
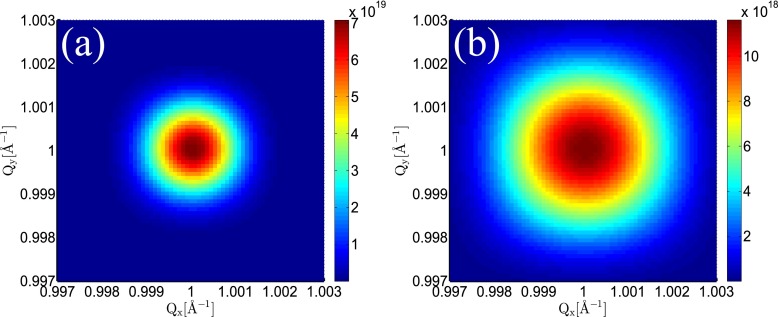
Contour plot for the Bragg spot of reflection (1 1 1) in the $Q_{z}=1\;{Å}^{-1}$ plane in reciprocal space: (a) Gaussian beam profile; (b) flattop beam profile.

The above findings are reflected by the time integrated signals that correspond to the situation one would encounter in an experiment (1D cut: Figs. 5(c) and 5(d); 2D cut: Fig. 6). Note that for the Gaussian spatial profile, there is only a small decrease of the signal compared to the ideal (no damage) case.

*Effect of the PBC approach on the x-ray scattering patterns.* In Section III B, we discussed that the PBC approximation overestimates ionization and atomic displacements, and therefore radiation damage throughout the sample. It means that the method gives an upper bound to the effect of radiation damage on the scattering patterns. A trivial lower bound is the case without any radiation damage.

## IV. CONCLUSIONS

We presented a methodology for the simulation of x-ray scattering patterns from serial femtosecond crystallography experiments with a high intensity x-ray beam. Our approach includes the simulation of radiation damage within the sample with the codes XMDYN and XATOM as well as the calculation of the patterns using the code XSINC. In the approach the crystal is divided into smaller units. The time evolution (the radiation damage process) of these units is calculated using periodic boundary conditions. Finally, a nanocrystal is assembled from the small units for the calculation of the patterns integrated over the pulse.

As a demonstration, we investigated spatial pulse profile effects on the Bragg peaks for a diamond nanocrystal. We found that if a Gaussian profile is used (assuming realistic XFEL parameters such as tight focus and ultrashort pulse duration), the time integrated signal intensity is reduced only by a small amount compared to the damage-free case. For a flattop profile, the decrease is much more significant. The intensity reduction is due to the change of the form factors caused by ionizations. In both cases, the width of the Bragg peak was connected to the size of the illuminated region in the crystal, but was not affected by damage. We analyzed the shortcoming of the periodic boundary condition approach. The method overestimates radiation damage in the interaction region, so it gives an upper bound to the effect of radiation damage on the patterns. In the future, the simulation method developed here is to be applied to more complex scenarios.
